# A Hybrid Scale-Up and Scale-Out Approach for Performance and Energy Efficiency Optimization in Systolic Array Accelerators

**DOI:** 10.3390/mi16030336

**Published:** 2025-03-14

**Authors:** Hao Sun, Junzhong Shen, Changwu Zhang, Hengzhu Liu

**Affiliations:** 1 College of Computer Science and Technology, National University of Defense Technology, Changsha 410073, China; sunhao1996@nudt.edu.cn (H.S.); shenjunzhong@nudt.edu.cn (J.S.); hengzhuliu@nudt.edu.cn (H.L.); 2 Key Laboratory of Advanced Microprocessor Chips and Systems, Changsha 410073, China; 3 Academy of Military Science, Beijing 100091, China

**Keywords:** systolic array, deep neural network, performance optimization, energy efficiency, accelerators

## Abstract

The rapid development of deep neural networks (DNNs), such as convolutional neural networks and transformer-based large language models, has significantly advanced AI applications. However, these advances have introduced substantial computational and data demands, presenting challenges for the development of systolic array accelerators, which excel in tensor operations. Systolic array accelerators are typically developed using two approaches: scale-up, which increases the size of a single array, and scale-out, which involves multiple parallel arrays of fixed size. Scale-up achieves high performance in large-scale matrix multiplications, while scale-out offers better energy efficiency for lower-dimensional matrix multiplications. However, neither approach can simultaneously maintain both high performance and high energy efficiency across the full spectrum of DNN tasks. In this work, we propose a hybrid approach that integrates scale-up and scale-out techniques. We use mapping space exploration in a multi-tenant application environment to assign DNN operations to specific systolic array modules, thereby optimizing performance and energy efficiency. Experiments show that our proposed hybrid systolic array accelerator reduces energy consumption by up to 8% on average and improves throughput by up to 57% on average, compared to TPUv3 across various DNN models.

## 1. Introduction

In recent years, artificial intelligence (AI) has achieved remarkable progress across various domains, including healthcare [1], autonomous driving [2], and robotics [3]. In the development of AI, convolutional neural networks (CNNs) and Transformer-based large language models (LLMs) have become the mainstream models of deep neural networks (DNNs) [4,5]. The commonality between these two mainstream models is the execution of numerous matrix multiplication operations, where the convolution operation in CNNs can be transformed into matrix multiplication through the im2col algorithm [6]. Systolic arrays are highly suited for matrix multiplication tasks [7]. Systolic array accelerators, exemplified by Google TPU [8] and Eyeriss [9], offer higher energy efficiency and throughput due to their data reuse characteristics and high parallelism [8,10], compared to CPUs and GPGPUs. However, as the computational demands and data requirements of DNNs grow exponentially, systolic arrays must scale accordingly to keep up with the evolving needs of DNN development.

As the scale of AI increases, one trend in the development of systolic array accelerators is the scaling up of individual systolic array sizes from 8 × 8 to 512 × 512 [11]. Larger systolic arrays can simplify on-chip interconnects while reducing the overhead associated with task partitioning and scheduling for matrix multiplication operations, resulting in higher throughput. This systolic array expansion method is referred to as the scale-up approach. Another trend in systolic array accelerators is the integration of multiple smaller systolic arrays [11]. Smaller systolic arrays offer greater flexibility and scalability, allowing for efficient task allocation when handling tasks with lower dimensionality, thereby reducing the waste of computational resources due to unnecessary zero-padding and minimizing energy consumption. The method involving multiple smaller systolic arrays is referred to as the scale-out approach. As various DNN models in AI continue to scale, there is no fixed rule for the development of task dimensions, which means that systolic array accelerators must evolve to handle a variety of computational tasks. However, the current development of systolic array accelerators is largely based on these two trends, each excelling in matrix multiplication computations with either fixed or variable dimensions [10]. To ensure both high energy efficiency and high performance across diverse tasks, the development of hybrid scale-up and scale-out systolic array accelerators is an effective strategy to address the varying characteristics of different computational tasks.

In this work, we propose a hybrid approach that integrates scale-up and scale-out systolic arrays to accelerate DNN tasks, aiming to improve performance and energy efficiency by adapting to the varying scales of matrix multiplication operations in DNNs. In multi-tenant application scenarios, we perform mapping space exploration to identify the systolic array modules that achieve the lowest energy consumption and highest throughput for different tasks, dynamically allocating tasks to the most suitable array module. During the mapping space exploration, we employ energy-first, throughput-first, and overall performance-first modes, allowing the hybrid systolic array accelerator to maximize performance and energy efficiency based on different user requirements. Across various CNN and LLM models, our proposed hybrid systolic array accelerator outperforms the uniform-scale systolic array accelerators in terms of both energy consumption and throughput. The primary contributions of this work are as follows:

- We propose a hybrid approach for constructing scale-up and scale-out systolic array accelerators.
- To meet different user objectives, we have implemented mapping space exploration for the hybrid systolic array accelerator, focusing on energy-first, throughput-first, and overall performance-first optimization.
- Our proposed hybrid systolic array accelerator reduces energy consumption by up to 8% on average and increases throughput by up to 57% compared to the TPUv3 configuration, respectively.
- Compared to the state-of-the-art scale-out accelerator SOSA [11], our hybrid systolic array accelerator achieves up to 5% higher throughput at the same energy consumption. With just a 1% increase in energy consumption, our proposed hybrid systolic array accelerator can boost throughput by up to 89% under the benchmarks in this work.

The remainder of the paper is organized as follows: Section 2 provides the background and reviews related work. Section 3 discusses the motivation behind the hybrid systolic array accelerator. Section 4 describes the method for constructing a hybrid accelerator that combines scale-up and scale-out systolic arrays. Section 5 presents the experimental results, highlighting the performance and energy efficiency improvements of the proposed hybrid systolic array accelerator. Finally, Section 6 concludes the paper.

## 2. Background and Related Work

### 2.1. Systolic Array Accelerators

**Figure 1:**
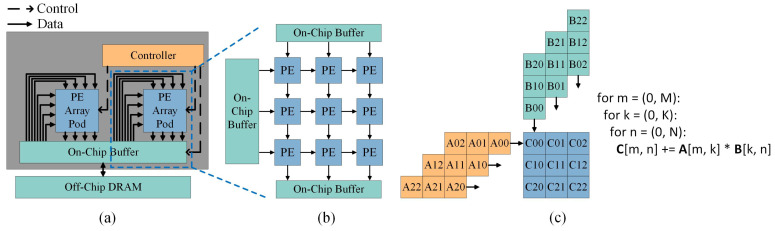
(**a**) Overview of the systolic array accelerator system. (**b**) Internal structure of the systolic array and its interconnection with the on-chip shared buffer. (**c**) Dataflow of the systolic array during matrix multiplication tasks.

The systolic array accelerator is an DNN accelerator that employs a systolic array as the core computational module for performing multiply-accumulate (MAC) operations. As depicted in Figure 1a, the primary components of the systolic array accelerator include both storage and computation modules. The computation module is primarily composed of the systolic array, which consists of numerous processing elements (PEs) arranged in a two-dimensional spatial configuration. These PEs are capable of executing MAC operations efficiently. The storage module is organized into three hierarchical levels: off-chip DRAM, on-chip shared buffers, and local buffers within each PE. Figure 1b illustrates the data transfer mechanism within the systolic array, where vertically and horizontally adjacent PEs exchange data over short distances through local buffers embedded within each PE. The PEs situated at the outermost periphery of the systolic array handle the data transfer with the on-chip shared buffer. Figure 1c demonstrates the dataflow for executing a matrix multiplication task on the systolic array. The accelerator executes computational tasks based on highly predictable dataflows, which reduces instruction overhead from the control module and minimizes the energy consumption and latency typically associated with frequent accesses to off-chip memory and on-chip global buffers.

### 2.2. Related Work

#### 2.2.1. Scalability and Flexibility in Systolic Array Design

Early designs like TPU [8] and Eyeriss [9] established the potential of systolic arrays for matrix multiplication, emphasizing fixed-scale architectures. To address diverse DNN workloads, researchers introduced reconfigurable designs such as SARA [12] (bypass paths for dynamic reconfiguration) and HeSA [13] (heterogeneous arrays for depthwise convolution). Gemmini [14] further advanced modularity through parameterizable systolic array generation.

Recent efforts in multi-pod architectures have optimized scalability. SOSA [11] proposed a multi-pod accelerator with Butterfly interconnects and custom data tiling, achieving 600 TeraOps/s and outperforming prior designs by 1.5×. Similarly, Maestro [15] explored 3D stacking for parallelism, while SCNN [16] integrated sparse computation across arrays. These works highlight the importance of balancing array granularity and interconnect efficiency, but none addressed hybrid scale-up/scale-out configurations.

#### 2.2.2. Energy Efficiency and Constraint-Aware Mapping

Energy optimization has been driven by tools like TENET [17], which modeled dataflows to maximize utilization, and AutoSA [18], which automated FPGA-based synthesis. However, idealized assumptions in prior works often ignored hardware constraints. FAMS [19] addressed this gap with a memory-centric mapping framework, incorporating communication-computation delays and multi-level memory capacities.

Dynamic workload allocation strategies further enhanced efficiency. RASA [20] optimized CPU-systolic integration via instruction overlap, while SMT-SA [21] used multithreading for sparse workloads. TensorLib [22] and FlexSA [23] enabled adaptive tensor mapping and sub-array reconfiguration, respectively. These approaches emphasized adaptability but lacked systematic integration with multi-pod architectures.

## 3. Motivation

The increasing computational and data demands of DNN models have driven the need for larger systolic array architectures. Two primary strategies exist for expanding systolic array configurations on a single chip: scale-up and scale-out approaches [10]. The scale-up approach involves enlarging a single systolic array by increasing its dimensions, while the scale-out method maintains fixed-size arrays but increases their number to enable parallel task execution. The scale-out approach leverages multiple smaller systolic arrays to optimize PE utilization within each array, resulting in reduced energy consumption. This efficiency arises from the ability to avoid redundant data transfers and computational operations, which would otherwise be necessary for zero-padding in large monolithic systolic arrays. However, for large-scale computational tasks, the scale-up approach with a larger single array typically achieves higher throughput, benefiting from greater internal parallelism, simplified control logic, and reduced communication overhead.

Given that both scale-up and scale-out systolic arrays offer distinct advantages in matrix multiplication operations, we adopted two key metrics, performance and energy efficiency, to compare their potential in DNN tasks. The first metric is the average energy consumption per MAC operation, calculated by dividing the dynamic power consumption during task execution by the total number of MAC operations. The second metric is throughput, defined as the average number of MAC operations completed per clock cycle for a given task. This is determined by dividing the total number of MAC operations by the task’s latency.

**Figure 2:**
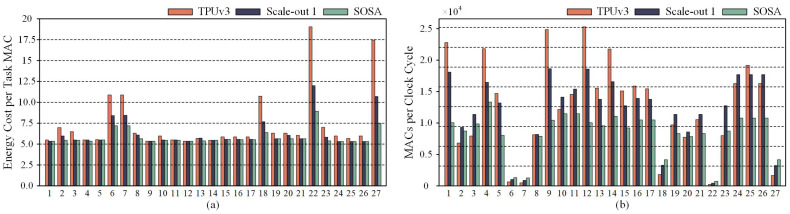
A comparison of relative energy consumption and throughput under various scale-up and scale-out systolic array configurations for the first four attention layers of Transformer, TinyBERT, T5, and the first five convolutional layers of AlexNet, NIN, and miniVGGNet: (**a**) the relative energy consumption under the mapping strategy that minimizes energy consumption; (**b**) the throughput under the mapping strategy that maximizes throughput.

We employed the TPUv3 configuration, featuring two pods of 128 × 128 systolic arrays, as our baseline. This setup was compared against the SOSA-provided configuration, which includes thirty-two pods of 32 × 32 systolic arrays [11], and the Scale-out 1 configuration, consisting of eight pods of 64 × 64 systolic arrays. We selected operational layers from common DNN models of LLMs and CNNs as benchmarks. The comparison results for these three systolic array accelerator configurations are shown in Figure 2.

Compared to TPUv3, the Scale-out 1 configuration and the systolic arrays from SOSA generally exhibit lower energy consumption. However, the TPUv3 configuration achieves higher throughput in nearly half of the cases. From the scale-up approach of TPUv3 to the scale-out configuration proposed by SOSA, these systolic array architectures face challenges in simultaneously achieving both low energy consumption and high throughput. Adopting a single systolic array construction strategy makes it difficult to ensure both high computational performance and energy efficiency. To strike a balance between performance and energy efficiency, it is essential to combine both scale-up and scale-out systolic array expansion strategies when designing a systolic array accelerator.

## 4. Hybrid Design of Scale-Up and Scale-Out Systolic Array Accelerators

In this work, we propose a hybrid method that integrates scale-up systolic arrays with scale-out systolic arrays within a single accelerator, aiming to enhance both the performance and energy efficiency of DNN tasks. Scale-up and scale-out systolic arrays are optimized for different computational characteristics, each providing distinct advantages for specific types of tasks. To fully exploit these advantages, we employ a dynamic mapping strategy that allocates DNN tasks to the most suitable scale-up or scale-out systolic arrays, depending on the task requirements. The proposed hybrid systolic array accelerator is specifically designed for multi-tenant environments, where it aims to improve performance and energy efficiency by selecting the appropriate array type, either scale-up or scale-out, based on the specific task at hand. To demonstrate the effectiveness of this approach, we present a case study using the TPUv3 configuration, as outlined in the motivation section.

**Figure 3:**
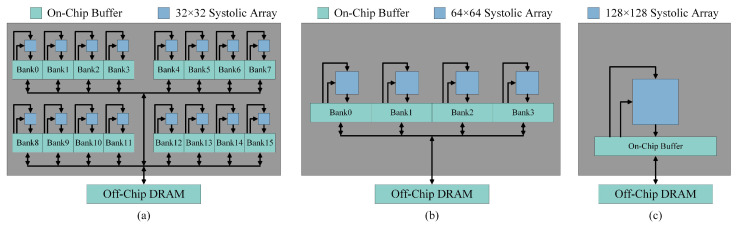
(**a**) Configuration of 16 pods of 32 × 32 scale-out systolic arrays. (**b**) Configuration of 4 pods of 64 × 64 scale-out systolic arrays. (**c**) Configuration of a single pod of a 128 × 128 scale-up systolic array.

Figure 3 illustrates the different scale-up and scale-out systolic array modules designed in this work. To simplify the management of interconnect networks and routing, and to enhance parallel processing capabilities, we evenly allocate on-chip shared buffers and bandwidth resources to each systolic array pod based on its size. Figure 3a shows the scale-out configuration with 16 pods of 32 × 32 systolic arrays. Figure 3b depicts the scale-up configuration derived from the setup in Figure 3a, where the size of each systolic array is increased and the number of systolic arrays is reduced. This configuration includes four pods of 64 × 64 systolic arrays. Figure 3c presents the scale-up configuration with a single pod of 128 × 128 systolic array. The different scale-out and scale-up systolic array modules shown in Figure 3a–c serve as the constituent components of the hybrid systolic array accelerator. In this case study, we consider three different types of hybrid systolic array accelerators: Hybrid 1, which combines 1 pod of a 128 × 128 array with 4 pods of 64 × 64 arrays; Hybrid 2, which combines 1 pod of a 128 × 128 array with 16 pods of 32 × 32 arrays; and Hybrid 3, which combines 4 pods of 64 × 64 arrays with 16 pods of 32 × 32 arrays.

We use mapping space exploration to determine the optimal mapping strategy for computational tasks on the hybrid systolic array accelerator. The mapping space exploration is guided by three distinct strategies: low-energy mode, high-throughput mode, and overall performance mode. The objective of the low-energy mode is to minimize the dynamic energy consumption associated with computation and communication during DNN task execution. In the high-throughput mode, the goal is to maximize the throughput of the systolic array modules in use. The overall performance mode aims to increase throughput while simultaneously reducing energy consumption. For the purpose of consistent comparison, we use energy consumption per task’s MAC count as the metric for the average energy required to execute each MAC operation in the low-energy mode. In the high-throughput mode, we use PE utilization as a fair metric to measure throughput across different numbers of PEs. In the overall performance mode, we derive a composite performance metric by dividing the energy consumption metric from the low-energy mode by PE utilization. A lower value of this composite performance metric indicates better overall performance. During mapping space exploration, we optimize the mapping strategy by minimizing the average energy consumption metric, maximizing the average PE utilization, and minimizing the composite performance metric.

In this work, we use the FAMS framework for mapping space exploration [19]. The FAMS framework is a specialized tool for design space exploration and mapping space exploration tailored to systolic array accelerators. Its memory-centric notation formalizes the representation of DNN task mappings onto systolic array accelerators, aiding in the establishment of the mapping space. Additionally, the FAMS framework includes a clock-accurate simulator that simulates hardware configurations and mapping strategies, providing metrics such as latency, energy consumption, throughput, and utilization. For mapping space exploration, we adopt an exhaustive search method to identify the optimal mapping strategy, along with corresponding metrics such as average energy consumption, average PE utilization, and the composite performance metric.

## 5. Experiments

### 5.1. Experimental Setup

#### 5.1.1. Benchmarks

We used multiple classical CNN and LLM models as benchmarks to compare the performance of the hybrid systolic array accelerators, including AlexNet [4], Network in Network (NIN) [24], VGGNet [25], GoogLeNet [26], ResNet [27], Transformer [5], TinyBERT4 [28], T5 [29], XLM [30], and GPT-3 [31].

#### 5.1.2. Simulation Tools, Experimental Methodology, and Metrics

As detailed in Section 4, we employed the FAMS framework as our experimental tool. The FAMS framework is specifically designed to aid in the design, optimization, and evaluation of DNN systolic array accelerators. In low-energy mode, we explore the mapping space for three types of scale-up and scale-out systolic array modules, identifying the most energy-efficient mapping strategies along with their corresponding PE utilizations. In high-throughput mode, we similarly explore the mapping space for these modules, determining the mapping strategies that yield the highest PE utilization and their corresponding energy consumption. In the overall performance mode, we explore the mapping space for these modules to identify the mapping strategies with the highest overall performance, along with their associated energy consumption, PE utilization, and composite performance metric.

In the following experiments, we compare the performance of our proposed hybrid systolic array accelerators with that of uniform scale systolic array accelerators, which match the array sizes of the TPUv3. The uniform scale systolic array accelerators include configurations identical to TPUv3, such as 2 pods of 128 × 128 systolic arrays, 8 pods of 64 × 64 systolic arrays, and 32 pods of 32 × 32 systolic arrays, as proposed by the state-of-the-art work SOSA. The hybrid systolic array accelerators consist of three different configurations: Hybrid 1, which combines 1 pod of a 128 × 128 systolic array with 4 pods of 64 × 64 systolic arrays; Hybrid 2, which combines one pod of a 128 × 128 systolic array with 16 pods of 32 × 32 systolic arrays; and Hybrid 3, which combines 4 pods of 64 × 64 systolic arrays with 16 pods of 32 × 32 systolic arrays.

### 5.2. Performance Comparison in Low-Energy Cost Mode

**Figure 4:**
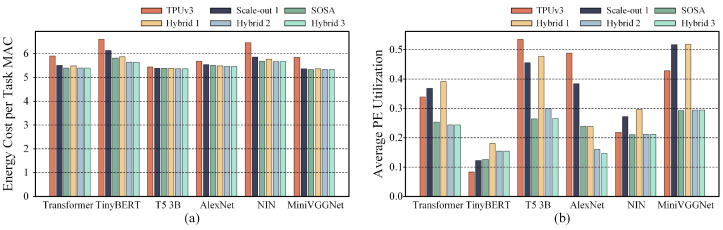
(**a**,**b**) Comparison of the average energy consumption and average PE utilization, respectively, between uniform-scale systolic array accelerators and three types of hybrid systolic array accelerators under mapping space exploration in low-energy mode.

We first compared the hybrid systolic array accelerators and the uniform scale systolic array accelerators in low-energy mode, with their energy consumption and PE utilization presented in Figure 4. As shown in Figure 4a, Hybrid 2 and Hybrid 3 achieve lower energy consumption. The energy consumption of Hybrid 1 is lower than that of TPUv3 and Scale-out 1 but slightly higher than that of SOSA. Figure 4b shows that, in low-energy mode, Hybrid 1 achieves the highest PE utilization for more than half of the tasks, while the PE utilization rates of Hybrid 2 and Hybrid 3 are comparatively lower.

As shown in Table 1, the performance and PE utilization of all compared systolic array accelerators across various DNN tasks were normalized to the results of TPUv3. The results indicate that Hybrid 2 and Hybrid 3 are the most effective in reducing energy consumption, achieving a reduction of 8% compared to TPUv3, which is equivalent to the SOSA configuration. Hybrid 1’s performance is the optimal, outperforming only TPUv3 and Scale-out 1 configurations. Under the lowest energy consumption mapping strategies, although Hybrid 2 and Hybrid 3 achieve the same energy consumption as SOSA, their average PE utilization rates are 4% and 3% higher, respectively, but still lower than those of TPUv3 and Scale-out 1. In contrast, although the energy reduction of Hybrid 1 is less pronounced than that of Hybrid 2 and Hybrid 3, it exhibits the highest PE utilization under the lowest energy consumption mapping strategy, which is 10% higher than the second highest, Scale-out 1. From these results, we observe a key pattern: when targeting low energy consumption, scale-up systolic arrays tend to have higher energy consumption but deliver significantly higher throughput, while scale-out systolic arrays achieve very low energy consumption at the expense of reduced average PE utilization.

### 5.3. Performance Comparison in High-Throughput Mode

**Figure 5:**
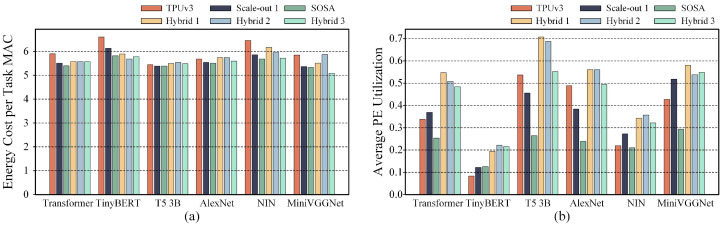
(**a**,**b**) Comparison of average energy consumption and average PE utilization, respectively, between uniform-scale systolic array accelerators and three types of hybrid systolic array accelerators under mapping space exploration in high-throughput mode.

Under the high-throughput mode, we focused on maximizing PE utilization during the mapping space exploration for all compared systolic array accelerators. Figure 5 presents a comparison of energy consumption and PE utilization under the high-throughput mode. As shown in Figure 5b, Hybrid 1, Hybrid 2, and Hybrid 3 all outperform TPUv3, Scale-out 1, and SOSA in terms of PE utilization. Notably, Hybrid 1 and Hybrid 2 achieve the highest PE utilization across all tasks. Figure 5a indicates that, under the optimal mapping strategies for high-throughput mode, the hybrid systolic array accelerators do not exhibit significant energy consumption advantages compared to the uniform scale systolic array accelerators for these DNN tasks.

As shown in Table 1, Hybrid 2 achieves the highest average increase in PE utilization across all DNN tasks, improving by 57% compared to TPUv3. In contrast, Hybrid 1 and Hybrid 3 show improvements of 55% and 46%, respectively, compared to TPUv3. Under the high-throughput mode, the hybrid systolic array architectures also result in lower energy consumption than TPUv3. However, the energy consumption of the hybrid systolic array accelerators is slightly higher than that of the SOSA configuration, which achieves the lowest energy consumption.

### 5.4. Performance Comparison Under High-Overall Performance Mode

**Figure 6:**
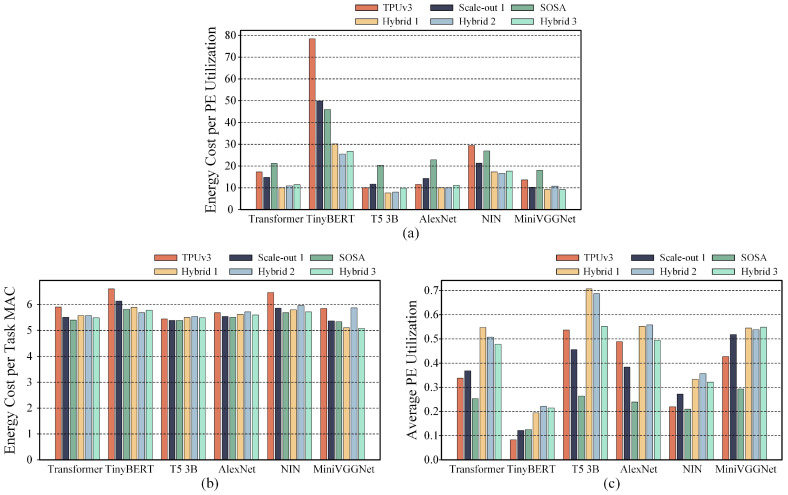
(**a**–**c**) Comparison of the composite performance metric, average energy consumption, and average PE utilization, respectively, between uniform-scale systolic array accelerators and three types of hybrid systolic array accelerators under mapping space exploration in high-overall performance mode.

We use the energy consumption per MAC operation during DNN task execution, divided by the average PE utilization, as a composite performance metric to evaluate the systolic array accelerators. A lower value of this composite metric indicates that the systolic array accelerator achieves both lower energy consumption and higher throughput. We performed mapping space exploration with the objective of minimizing this composite performance metric. The corresponding values for the composite performance metric, energy consumption, and PE utilization are shown in Figure 6.

In Figure 6a, it is evident that the three hybrid systolic array accelerators outperform the uniform scale systolic array accelerators in terms of composite performance across various DNN tasks. Each hybrid systolic array accelerator offers unique advantages for different DNN tasks, with no single configuration consistently providing the optimal composite performance. Figure 6b shows that, under the optimal composite performance strategy, the hybrid systolic array accelerators do not exhibit a significant advantage in terms of energy consumption. However, as shown in Figure 6c, the throughput of the hybrid systolic array accelerators is consistently higher than that of the uniform scale systolic array accelerators. Therefore, we conclude that the hybrid systolic array accelerators achieve more substantial improvements in throughput compared to the uniform scale systolic array accelerators, while the improvement in energy efficiency is relatively modest.

As shown in Table 1, under the currently used composite performance metric, Hybrid 1 achieves the best overall performance, followed by Hybrid 2 in second place and Hybrid 3 in third. However, compared to the uniform scale systolic array accelerators, all three hybrid systolic array accelerators outperform them in terms of overall composite performance.

### 5.5. Case Study on Expanded Hardware Configurations and Tasks

**Figure 7:**
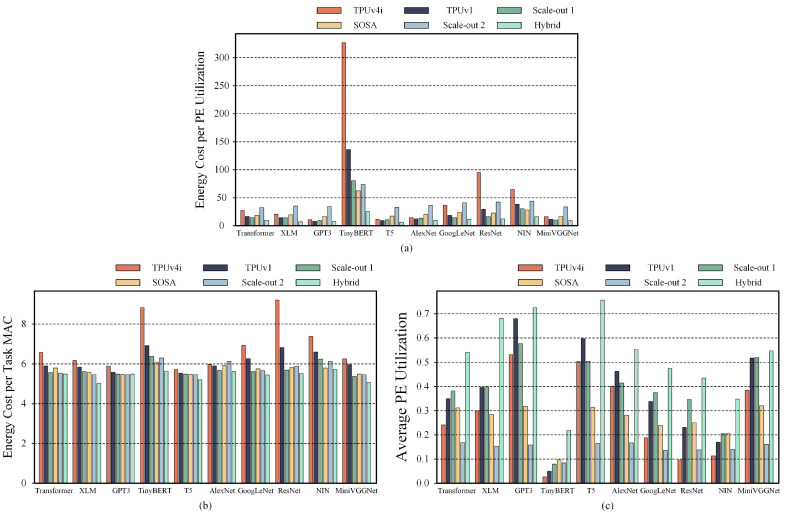
(**a**–**c**) Comparison of the composite performance metric, average energy consumption, and average PE utilization, respectively, between uniform-scale systolic array accelerators and hybrid systolic array accelerators under mapping space exploration in high-overall performance mode.

To explore the versatility of our hybrid systolic array architecture, we compared the performance of various systolic array configurations across a broader range of DNN tasks, using the TPUv4i configuration as a benchmark. TPUv4i consists of four pods of a 128 × 128 systolic array, and its systolic array size configuration is similar to that of TPUv1, which includes a single pod of a 256 × 256 systolic array [11]. In addition, we compared the 32 × 32 systolic array pods proposed by SOSA, the 64 × 64 Scale-out 1 systolic array pods, and the 16 × 16 Scale-out 2 systolic array pods. For our hybrid systolic array architecture, we used four different systolic array configurations: 1 pod of a 128 × 128 systolic array, 4 pods of a 64 × 64 systolic array, 16 pods of a 32 × 32 systolic array, and 64 pods of a 16 × 16 systolic array. In addition to the six DNN tasks used in previous experiments, we included several other tasks, such as XLM, GPT-3, GoogLeNet, and ResNet. Figure 7a–c show the comparative results for the composite performance metric, the average energy consumption per MAC operation, and the average PE utilization, respectively.

The composite performance of the hybrid systolic array accelerators consistently outperforms that of other uniform scale systolic array accelerators across all tasks. Correspondingly, the energy consumption of the hybrid systolic array accelerators is lower than that of the other accelerators, though the difference is not highly significant. In terms of PE utilization, the hybrid systolic array accelerators show a substantial improvement in most tasks.

## 6. Conclusions

In this study, we introduced a hybrid approach for building systolic array accelerators that integrates both scale-out and scale-up strategies. This hybrid systolic array accelerator merges the benefits of scale-up and scale-out configurations, thereby ensuring high performance and energy efficiency for DNN tasks, complemented by adaptable mapping strategies. In environments with multiple tenants, these dynamic mapping strategies efficiently allocate DNN tasks to the systolic array pod that delivers optimal performance and energy efficiency. Across a range of DNN tasks, our proposed hybrid systolic array accelerator demonstrates superior performance compared to TPUv1, TPUv3, TPUv4i, and the state-of-the-art systolic array architecture SOSA, excelling in both throughput and energy efficiency.

## Figures and Tables

**Table 1:** A comparison of energy consumption, PE utilization, and the composite performance metric under low-energy mode, high-throughput mode, and overall performance mode.

Mode	Metric	TPUv3	Scale-Out 1	SOSA	Hybrid 1	Hybrid 2	Hybrid 3
Low-Energy	Energy	1	0.94	0.92	0.93	0.92	0.92
	PE utilization	1	1.11	0.81	1.21	0.85	0.84
High-Throughput	Energy	1	0.94	0.92	0.96	0.96	0.93
	PE utilization	1	1.11	0.81	1.55	1.57	1.46
High-Overall Performance	Energy	1	0.94	0.92	0.93	0.96	0.93
	PE utilization	1	1.11	0.81	1.53	1.57	1.46
	Composite performance	1	0.90	1.34	0.65	0.67	0.71

## Data Availability

The datasets used and/or analyzed during the current study are available from the corresponding author upon reasonable request.
